# Disproportionately Low Albumin and High Neutrophil-to-Lymphocyte Ratio in Small Bowel Adenocarcinoma Patients With Long-Duration Crohn's Disease

**DOI:** 10.14309/ctg.0000000000000553

**Published:** 2022-11-30

**Authors:** Ido Veisman, Amit Oppenheim, Zuheir Sub laban, Ariel Kenig, Offir Ukashi, Einat Shacham-Shmueli, Tal Engel, Uri Kopylov, Shomron Ben-Horin, Alon Lang

**Affiliations:** 1 Department of Gastroenterology, Sheba Medical Center, Tel Hashomer, Israel;; 2 Sackler School of Medicine, Tel-Aviv University, Tel-Aviv, Israel;; 3 Department of Internal Medicine A, Sheba Medical Center, Tel Hashomer, Israel;; 4 Department of Medicine, Hadassah Medical Center, Jerusalem, Israel;; 5 Faculty of Medicine, the Hebrew University, Jerusalem, Israel;; 6 Oncology Department, Sheba Medical Center, Tel Hashomer, Israel.

## Abstract

Although Crohn's disease (CD) is a known risk factor of small bowel adenocarcinoma (SBA), early diagnosis remains a significant clinical challenge. Identification of biomarkers for SBA may lead to early detection.

**METHODS:**

This is a retrospective study comparing albumin levels and neutrophil-to-lymphocyte ratio (NLR) of patients with long-standing CD who underwent small bowel resection with and without malignancy.

**RESULTS:**

Forty-two patients with CD were included in this study (11 with SBA). Median NLR before surgery was 8.5 (interquartile range 6.2–31.3) in patients with SBA and 3.8 (interquartile range 2.8–5.3) for patients without SBA (*P* < 0.05). Mean albumin levels before surgery were significantly lower among patients with SBA compared with patients without SBA (2.6 ± 0.6 g/dL vs 3.5 ± 0.6 g/dL, respectively, *P* < 0.05), despite patients with SBA being under longer total parenteral nutrition treatment duration.

**DISCUSSION:**

CD patients with SBA diagnosis have increased NLR and lower albumin before surgery compared with CD patients without detection of SBA.

## INTRODUCTION

The chronic inflammatory environment in the gastrointestinal (GI) tract among patients with inflammatory bowel disease is a known risk factor of GI malignancy. In a previous population-based study, the risk of small bowel adenocarcinoma (SBA) was increased by 67-fold in patients with Crohn's disease (CD) compared with non-IBD patients (1). Moreover, among patients with CD, SBA was found to be the most common small bowel malignancy, diagnosed at an younger age compared with the general population, with 7-fold higher risk of mortality (2). The clinical presentation for SBA is nonspecific, and the diagnosis is mostly obtained postoperatively after resection of an obstructed small bowel segment and often without a priori clinical suspicion of neoplasia. Owing to these difficulties, diagnosis is commonly made at advanced stages (3,4). Neutrophil-to-lymphocyte ratio (NLR) is a known marker of inflammation. It is also a predictor of poor outcomes and relapse risk in diagnosed cancer, including GI malignancies (5). Decreased albumin levels have been linked to malignancy as well. Therefore, this study examined a possible association between albumin, NLR, and diagnosis of SBA in patients with long-term (>10 years) CD.

## METHODS

This retrospective case-control study included adult (older than 18 years) patients with CD who underwent small bowel resection after at least 10 years from initial diagnosis between 2008 and 2020 at the Sheba Medical Center. Through file review, only cases of small bowel carcinoma were included in the malignancy group. Patients with CD (>10 years) who underwent small bowel resection without malignancy served as controls in a 1:3 ratio. Owing to missing data, 2 patients from the no-malignancy group were excluded from the final analysis.

Baseline characteristics and CD-related features were collected from electronic medical records. All available albumin and NLR levels were collected from a 3-month period before surgery. Total parenteral nutrition (TPN) treatment duration was obtained for each patient over the 1-year period before surgery. Data were tested using the Shapiro-Wilk test for normality. For normally distributed data, mean and SD were calculated. Similarity between independent groups was tested using the Levene test for equality of variances and *t* test for equality of means. For non-normally distributed data, median and interquartile range (IQR) were calculated, and equality between groups was tested using an independent sample median test. Alpha was set at 0.05 for all tests. All analyses were performed using IBM-SPSS version 25.

## RESULTS

Eleven patients with CD diagnosed with SBA (malignancy group) were included. All these patients had CD involving the small bowel and diagnosed with SBA based on histopathological examination of surgical specimens, after a small bowel resection.

Baseline characteristics are presented in Table 1. The median age was 55 (IQR 44–65) years in the malignancy group and 37 (IQR 31–47) years in the no-malignancy group (*P* value <0.05). No significant difference in CD location or behavior was found between groups, except for a trend toward stricturing disease in malignancy vs no-malignancy cases (81.8% vs 45.2%, respectively, *P* value = 0.075). Mean albumin levels 3 months before surgery were significantly lower in the malignancy group (2.6 g/dL vs 3.5 g/dL_,_ *P* value <0.05) while this group was treated with TPN for significantly longer duration (2 vs 0 months, *P* value <0.05). Median NLR levels were significantly higher in the malignancy vs no-malignancy group (3.8 vs 8.5, respectively, *P* value <0.05). The number of hospitalizations during 1-year period before surgery was significantly higher when cancer was subsequently detected (Table 2).

**Table 1.** Patient characteristics

Parameter	No malignancy (n = 31)	Malignancy (n = 11)	Statistical significance for difference between groups^a^
Male, n (%)	19 (61.3%)	8 (73.7%)	0.717
Median age (q25-q75)	37 (31–47)	55 (44–65)	**0.005**
Median disease duration (q25-q75) (yr)	16 (13–20)	25 (10–30)	0.483
Crohn's disease location			
Ileal	16 (53.3%)	6 (54.5%)	0.945
Ileocolonic	14 (46.7%)	5 (45.5%)	0.945
Crohn's disease behavior			
Stricturing	14 (45.2%)	9 (81.8%)	0.075
Penetrating	16 (51.6%)	4 (36.4%)	0.384
Nonstricturing and nonpenetrating	4 (12.9%)	1 (9.1%)	1.000

a Statistical significance was calculated according to data characteristics by the Pearson χ^2^ test, Fisher exact test (2-sided), or independent samples median test.

**Table 2.** Group characteristics before surgery

	No malignancy (n = 31)	Malignancy (n = 11)	Statistical significance for difference between groups^a^
Albumin level during 3 mo before surgery—mean ± SD (g/dL)	3.5 ± 0.6	2.6 ± 0.6	**0.000**
Neutrophil-to-lymphocyte ratio during 3 mo before surgery—median (q25-q75)	3.8 (2.8–5.3)	8.5 (6.2–31.3)	**0.004**
Patients on TPN, n (%)	7 (22.5%)	8 (72.7%)	**0.0008**
Total parenteral nutrition treatment duration in mo during 1 yr before surgery—median (q25-q75)	0.0 (0.0–0.0)	2.0 (0.0–4.0)	**0.009**
No. of hospitalizations during a 1-year period before surgery—median (q25-q75)	2.0 (0.0–3.0)	3.0 (2.0–4.0)	**0.048**

Significance for bold entries was *P* value ≤ 0.05.

a Statistical significance was calculated according to data distribution by the *t* test or independent samples median test.

TPN, total parenteral nutrition.

## DISCUSSION

Since first described in 1956 (6), numerous studies showed an increased risk of SBA among patients with CD. However, SBA detection is still a significant challenge, and the diagnosis is made mostly at an advanced stage. Owing to low sensitivity, endoscopic screening surveillance is not recommended for SBA in patients with long-term CD (7). In addition, circulating carcinoembryonic antigen was found to be increased in up to 38% of active patients with CD (8); hence, it cannot be used as a malignancy screening tool among these patients. Magnetic resonance enterography and video capsule endoscopy could potentially detect SBA, yet distinction between inflammatory stenosis and malignancy remains challenging with significant economic implications, thus not recommended. Therefore, identification of markers for SBA among patients with CD is of crucial importance.

NLR is a well-known marker of inflammation. Previous studies demonstrated that NLR is a strong and independent risk indicator for mortality in the general population and among patients suffering from malignancies, including GI malignancies (5). Moreover, high NLR levels were found to be associated with the risk of detecting malignancies among patients who underwent prostate biopsy (9). We found that mean NLR levels were significantly increased among patients with CD who underwent small bowel resection with SBA detected in histopathology in comparison with those for whom carcinoma was ruled out.

Albumin is the most abundant protein in the human serum, and hypoalbuminemia is a common manifestation among oncologic patients. Moreover, serum albumin level is an independent prognostic factor in several cancers, including GI malignancies (10). However, albumin level does not serve as a marker, and data regarding albumin levels and small bowel malignancies among patients with CD are scarce. In this study, a comparison between albumin levels 3 month before surgery showed that albumin levels in the malignancy group were significantly lower compared with the no-malignancy group. Despite higher rates of TPN treatment initiated in part due to low albumin levels within 1 year before surgery among patients with SBA, presurgery lower albumin levels were still found in patients with SBA compared with non-SBA patients. The total number of hospitalizations during the year presurgery was significantly higher in the malignancy group; however, it should be noted that this was a small numerical difference.

According to previous studies, the usual age of SBA diagnosis among patients with CD is 45–55 years, younger than SBA *de novo* which is usually diagnosed between 60 and 69 years. In our study, the median age for SBA diagnosis was 55 (IQR 44–65) years—older than previously published mean age for diagnosis and on the upper age range of other previous reports (3). This difference might be explained by delay in clinical suspicion, better control of the underlying inflammatory disease (delayed need for surgery), or other inherent differences between our cohort and previous cohorts.

In conclusion, this study demonstrates for the first time an association between marked hypoalbuminemia and an increased NLR with the diagnosis of small bowel malignancy among patients with CD. If these findings are corroborated by large additional studies, NLR and disproportionate hypoalbuminemia may become important diagnostic clues for SBA in long-standing CD.

## CONFLICTS OF INTEREST

**Guarantor of the article:** Ido Veisman, MD.

**Specific author contributions:** I.V., A.O., U.K., S.B.-H., and A.L.: conceptualization. T.E., Z.B.L., O.U., and A.K.: acquisition of data. I.V., A.O., E.S.-S., and A.L: analysis and interpretation of data.

**Financial support:** None to report.

**Potential competing interests:** U.K. received speaker and advisory fees from AbbVie, Janssen, Medtronic, MSD, and Takeda; research support from Takeda, Medtronic, and Janssen; and consulting fees from Takeda and CTS. S.B.-H. received consulting and advisory board fees and/or research support from AbbVie, MSD, Janssen, Takeda, Pfizer, GSK, Galmed Pharmaceuticals, and CellTrion.
